# An ecological study of the link between the risk of most frequent types of cancer in Poland and socioeconomic variables

**DOI:** 10.1007/s00038-018-1082-x

**Published:** 2018-03-05

**Authors:** Katarzyna Orlewska, Andrzej Sliwczynski, Ewa Orlewska

**Affiliations:** 1 Medical University of Warsaw, Warsaw, Poland; 2 Medical University in Lodz, Lodz, Poland; 3 National Health Fund, Warsaw, Poland; 4 Faculty of Medicine and Health Sciences, The Jan Kochanowski University in Kielce, Al. IX Wieków Kielc 19, 25-317 Kielce, Poland

**Keywords:** Cancer risk, Poverty, Social isolation, Social capital, Religiousness

## Abstract

**Objectives:**

To assess the link between the risks of most frequent cancer sites in Poland and selected socioeconomic variables that potentially affect health outcomes throughout the life course.

**Methods:**

This is a cross-sectional ecological study. Incidence of lung, breast, and colon cancer by voivodeships in 2014 was calculated based on Polish National Cancer Registry. Socioeconomic variables in individual voivodeships were assessed based on Polish Social Cohesion Survey for 2015. Spearman’s rank correlation coefficient was used to test the association of incidence rates and socioeconomic variables. The significance level was set at *p* < 0.05 (two-tailed tests).

**Results:**

Statistically significant negative correlation exists between: (1) friend-/neighbour-based social capital and colon and breast cancer, (2) association-based social capital and lung cancer, (3) high religiousness and lung and breast cancer, and (4) income poverty and breast cancer. Statistically significant positive correlation exists between: (1) social isolation, living conditions poverty, poverty resulting from the lack of budget balance, and lung cancer; (2) low/no involvement in religious activity and lung and breast cancer.

**Conclusions:**

Our findings support public health concerns over the implication of socioeconomic environment for cancer.

## Introduction

Cancer is one of the major contributors to health burden worldwide. In 2012 14.1 million new cases and 8.2 million deaths were reported. Lung, breast and colorectal were the most frequently diagnosed cancers (Ferlay et al. 2015). Central and Eastern Europe is characterized by highest incidence and mortality rates of lung cancer (age-standardized rate 53.4 and 47.6, respectively) and highest mortality rate of colorectal cancer (age-standardized rate 20.3 among men and 10.11 among women) (Ferlay et al. 2015). Lung cancer is the prevailing cause of death and comprises a major social issue by all of malignancies occurring in Poland. Both morbidity and mortality due to lung cancer are increasing among the female population and steadily decreasing among men, most of all as a result of tobacco control in Poland over the past decade (Zatonski et al. 2015). Breast cancer accounts for 22% of all malignant neoplasms and is the second leading cancer-related death cause among Polish women. Between 1995 and 2014 breast cancer incidence rate showed 1.5-fold increase, while the mortality rate of breast cancer is gradually decreasing (Zatonski et al. 2015). Colon cancer incidence in Poland presented constant rises in the last two decades, but from the beginning of the 21st century the increase of colon cancer mortality has stopped among men and reversed among women (Zatonski et al. 2015). Trends in the mortality due to breast and colon cancer result from a variety of factors, including national screening programmes introduced in Poland in 2006. In general the mortality time trends for the main cancer sites in Poland are similar to those observed in other EU countries. However, the epidemiological transformation in cancer mortality in Poland is delayed with respect to Western European countries.

Having knowledge of the impact malignancies have on public health, it is essential to improve the understanding of which factors at which point are relevant to the disease. The multifactorial nature of cancer indicates that it is caused by a combination of environmental, genetic, lifestyle and socioeconomic factors developing over the lifetime of an individual. Established cancer risk factors include: tobacco and alcohol use, unhealthy diet, overweight/obesity, infections, reproductive factors, physical inactivity, selected occupational and environmental agents (Parkin et al. 2011; Danaei et al. 2005; Boffetta et al. 2009). Despite conceptual acknowledgment of the importance of social environment for cancer (Lynch and Rebbeck 2013; Gomez et al.^2015^), socioeconomic factors have rarely been considered in cancer research. Results from studies conducted in the last 20 years suggest the correlation between lung cancer risk and adult socioeconomic position (Power et al. 2005; Strand and Kunst 2007; De Kok et al. 2008), or with degree of disadvantage during one’s lifetime (Regidor et al. 2003; Galobardes et al. 2004; Næss et al. 2005; Lawlor et al. 2006). Stomach cancer has long been associated with social circumstances during infancy and adolescence (Power et al. 2005; Regidor et al. 2003; Galobardes et al. 2004, Næss et al. 2005; Lawlor et al. 2006), and breast, cervical, and colorectal cancer—with early social circumstances (De Kok et al. 2008; Regidor et al. 2003; Næss et al. 2005). A reversed association between cancer incidence and social deprivation has been recognized for malignant melanoma, breast, and prostate cancer (National Cancer Intelligence Network 2014).

Recently published studies have presented a strong association between higher social support and improved cancer treatment results, especially for breast cancer incidence and prognosis (Kroenke et al. 2017; Pinquart and Duberstein 2010; Beasley et al. 2010; Lutgendorf et al. 2012; Ikeda et al. 2013; Hinzey et al. 2016). In addition, some data suggest that social isolation and greater neighbourhood poverty are independently associated with an increased cancer risk and higher mortality (Fleisch et al. 2017; Conroy et al. 2017). Scientists have identified three main pathways through which poverty and social support may affect health: behavioural, psychological and physiological mechanisms (Berkman and Glass 2014; Hawkley and Cacioppo 2010). As all of these mechanisms interact with each other, a precise assessment of the social environment should not be limited to any specific indicator (e.g., education, profession, income, employment), but should acknowledge the social environment as a whole.

In Poland socioeconomic dissimilarities in health (e.g., cancer incidence) have rarely been analysed. Only one study, performed in the Opole province, found statistically significant positive correlation between unemployment rate and lung cancer incidence rates among male population (Chawińska et al. 2013). These results are consistent with the most recent reports which state that the more deprived area, the higher the burden of disease.

The aim of our cross-sectional ecological study was to assess the link between the risks of most frequent cancer sites in Poland and selected socioeconomic variables: social isolation, various social capital types, poverty, involvement in church/religious activity, attitude towards faith. These variables were selected because they potentially affect health outcomes throughout the life course. Understanding the role of these factors as determinants of cancer risk may offer opportunities for intervention in reducing disparities in cancer incidence. To this day no studies on this topic have been conducted in Poland. We chose voivodeship as the unit of analysis because the research question was formulated at the area-level and the main construct investigated (socioeconomic determinants) is conceptualized as an area-level attribute. Socioeconomic determinants are conceptualized as a group attribute that affects all individuals living within the community and the interest is drawing inferences regarding differences between areas.

## Methods

### Measurement of cancer risk by voivodeships

The cancer risk in each of 16 Polish voivodeships was calculated as incidence rates and expressed as the number of newly registered cases of cancer in 2014 per a population of 100,000. The absolute numbers of newly registered cases of lung, breast and colon cancer in Poland by voivodeships were retrieved from the Polish National Cancer Registry (NCR). NCR is a principal data source for cancer incidence in the Polish population. Healthcare providers are legally obliged to provide annual reports to NCR about every case of cancer diagnosed within the Polish population. Principles of data collecting and institutions responsible for cancer registries in Poland are specified in two acts: the Act on Public Statistics and the Act on the Health Care Information System and an ordinance issued on the basis of the said Act. The unit responsible for the registry is the National Cancer Registration Office, which is an organizational unit of the Cancer Centre and Institute of Oncology. New cases of cancer are collected via specific cancer registration forms (MZ/N-1a form) and each voivodeship is obliged to deliver the data to a shared NCR database. The data is validated using rules enforced to the system. An online database is available at http://onkologia.org. Data storage enables analysis of cancer incidence and mortality by type of cancer (classified according to ICD-10), age, sex and voivodeship, as well as creating custom reports. The average completeness of the Cancer Registry in Poland has been continuously improving and is currently estimated to be 94% (Cancer control strategy for Poland 2015–2024, 2014).

### Measurement of area-based socioeconomic status

Area-based socioeconomic measurements were retrieved from the Polish Social Cohesion Survey for the year 2015 (Bieńkuńska et al. 2017). This survey covered the entire Polish population and presented selected measures at a voivodeship level to illustrate territorial diversity of material situation, experienced poverty and social relationships. The survey sample comprised 27,117 dwellings, selected randomly using a two-stage stratified sampling scheme. The sample was drawn using the sampling frame established on the basis of the TERYT system, i.e., National Official Register of Territorial Division of the Country. Census areas served as first-stage sampling units, while second-stage involved drawing the dwellings sample. The share of refusals amounted to 26%. All methodological details have been published elsewhere (Verger et al. 2015; Bieńkuńska et al. 2017). Definitions of the key socioeconomic variables analysed in the study are presented in Table 1.

**Table 1:** Definitions of indicators used in the study (according to the Polish Social Cohesion Survey)

Thematic area	Indicator	Definition
Social contacts	Association-based social capital	% of persons aged 16 or more who declared their involvement in at least one organisation, association, or formal group
	Neighbour-based social capital	% of persons aged 16 or more who declared visiting their neighbours, spending time together, or doing various favours for each other
	Family based social capital	% of persons aged 16 or more who declared contacts with family members during which they were able to get material and spiritual support
	Social isolation	% of persons aged 16 or more for whom low (or zero) intensity of social contacts with persons from outside their household was observed. The social isolation was assessed on the basis of an aggregate indicator of social contacts with family, neighbours, friends, and colleagues, and the degree of involvement in social life and various organizational activities. The values of the indictor ranged from 0 (a “strongly socially isolated” person) to 10 (a “strongly socially integrated” person). The social isolation threshold was set at 3
Religious activity	Involvement in religious activity	Level of personal involvement in the social activity of a church/religious organisation of persons aged 16 or more based on 9 questions corresponding to the activity types and the sense of attachment. The categories of involvement are: (1) outside the church; (2) no involvement; (3) low level of involvement; (4) medium level of involvement; (5) high level of involvement
	Religiousness	The attitude towards faith was assessed by estimation of % of persons aged 16 or more who declared to be “profound believers”, “believers”, “hesitant/searching”, “neutral”, and “nonbelievers”
Poverty	Income poverty	% of households in which the monthly equivalised income at household’s disposal (within 12 months preceding the survey) was lower than the value regarded as the poverty threshold. The poverty threshold was assumed at 60% of the median equivalised income, i.e., income comparable between households with different demographic structures
	Living conditions poverty	% of households in which at least 10 indications of poor living conditions were observed, based on the list of 30 symptoms concerning the dwelling quality, the provision of durable consumer goods, and the deprivation of various types of consumer needs
	Poverty in terms of the lack of budget balance	% of households which were considered poor in terms of “inability to deal with their budget”, i.e., in which at least 4 out of 7 symptoms were identified, including both the subjective opinions of households on their material status, and the facts testifying to budget difficulties faced by the household

### Data analysis

The Spearman’s rank correlation coefficient (rS) was used to examine the link between incidence rates of particular types of cancer and socioeconomic determinants. A significance level of *p* < 0.05 was applied (2-tailed tests). The effect of potential confounders such as sex, age and completeness of cancer registration distribution across regions was accounted for by Spearman’s partial rank correlation. The basis for the evaluation of the completeness of cancer registration in particular voivodeships was the ratio of mortality to incidence (M/I–Index) calculated for each cancer site in the same period of time (Zanetti et al. 2015).

## Results

### Regional diversity of most common cancer incidence by voivodeships

The ranking of voivodeships according to incidence rates of particular cancers differs considerably (Table 2). The highest incidence rate of lung cancer was observed in Kuyavian-Pomeranian (80/100,000), the lowest—in Podlaskie (40/100,000), and in 9 voivodeships the value was higher than mean for entire Poland (57/100,000). The highest incidence rate of breast cancer was noted in Lodz (51/100,000), the lowest in Lublin, Podlaskie and Warmian-Masurian (39/100,000) and in 5 voivodeships the value was higher than mean for entire Poland (45/100,000). For colon cancer the highest incidence rate was in Kuyavian-Pomeranian (34/100,000), the lowest in Masovian (21/100,000) and in 6 voivodeships the value was higher than mean for entire Poland (27/100,000).

**Table 2:** Regional diversity of incidence of the most frequent cancer sites by voivodeships

Voivodeship	Lung cancer incidence	Breast cancer incidence	Colon cancer incidence
Lower Silesian	65	49	32
Kujavian-Pomeranian	80	47	34
Lublin	53	39	27
Lubusz	60	44	26
Lodz	56	55	28
Lesser Poland	48	41	24
Masovian	46	44	21
Opole	60	42	29
Subcarpathian	45	40	25
Podlaskie	41	39	24
Pomeranian	66	47	28
Silesian	60	44	26
Swietokrzyskie	65	40	29
Warmian-Masurian	73	39	26
Greater Poland	54	52	30
West Pomeranian	66	49	26

Incidence is expressed as the number of new cases of cancer registered in 2014 per a population of 100,000. Data obtained from the Polish National Cancer Registry (NCR); online database is available at http://onkologia.org

### Socioeconomic indicators for voivodeships

Social isolation, social capital, religious activity and poverty in individual voivodeships is presented in Table 3. The voivodeships with high value of the social isolation indicator included West Pomeranian (14%), Lodz (11%), Kuyavian-Pomeranian, Lubusz, Swietokrzyskie, and Warmian-Masurian (10%), lowest indicator values (5%) were recorded in Lublin, Podlaskie and Subcarpathian.

**Table 3:** Selected socioeconomic indicators for voivodeships: percentage of people socially isolated, percentage of people with high level of various social capital types, percentage of people with low/no involvement in religious activity, percentage of people declaring to be “profound believers”, and percentage of people affected with various types of poverty

Voivodeship	High level of social isolation (%)	High level of social capital			/no involvement in religious activity (%)	High religiousness (%)	High poverty		
		Association-based (%)	Family-based (%)	Friend- and neighbour-based (%)			Income (%)	Living conditions (%)	In terms of dealing with the household budget (%)
Lower Silesian	7	19	26	23	47	10	11	8	10
Kujavian-Pomeranian	10	20	24	21	44	7	17	8	12
Lublin	5	24	31	25	24	14	27	10	11
Lubusz	10	21	27	24	50	6	11	10	15
Lodz	11	14	29	23	49	7	15	11	15
Lesser Poland	8	20	31	34	26	12	15	8	9
Masovian	8	22	28	27	43	12	11	8	10
Opole	9	25	31	28	33	16	11	8	8
Subcarpathian	5	21	31	33	17	15	21	8	6
Podlaskie	5	31	24	31	26	15	17	5	7
Pomeranian	8	16	28	28	39	8	13	9	14
Silesian	8	19	30	25	44	10	9	7	12
Swietokrzyskie	10	16	34	32	34	11	24	11	11
Warmian-Masurian	10	13	25	31	54	8	17	12	14
Greater Poland	7	20	28	24	36	11	13	6	9
West Pomeranian	14	19	19	24	56	7	16	12	18

Data obtained from the polish social cohesion survey for the year 2015 (Bieńkuńska et al. 2017)

Voivodeships showed wide disparities in social capital levels. The lowest ratio for high degree of association-based social capital was recorded in Pomeranian, Swietokrzyskie (16%), Lodz (14%) and Warmian-Masurian (13%), whereas the highest in Podlaskie (31%), Opole (25%) and Lublin (24%). The share of persons with high level of familial capital was lowest in West Pomeranian (19%) and highest in Swietokrzyskie (34%), Opole (31%), Lesser Poland (31%), Subcarpathian (31%), Lublin (31%). The lowest value for the indicator for high level of friends- and neighbours-based social occurred in Kuyavian-Pomeranian (21%), Lodz and Lower Silesian (23%), the highest in Lesser Poland (34%), Subcarpathian (33%) and Swietokrzyskie (32%).

The attitude to faith varied from one voivodeship to another. Percentage of people who declared to be “profound believers” was highest in Opole (16%), Subcarpathian (15%), Podlaskie (15), Lublin (14%), and lowest—in Lubusz (6%), Lodz (7%), Kuyavian-Pomeranian (7%), and West-Pomeranian (7%). In 7 voivodeships the percentage of religiously uncommitted and nonclerical people is lower than the mean for entire Poland (39%), voivodeships with the topmost percentage of religiously uncommitted inhabitants are West Pomeranian (56%), Warmian-Masurian (54%) and Lubusz (50%).

Income poverty affects inhabitants of Lublin (27%), Swietokrzyskie (24%) and Subcarpathian (21%) more often, whereas those residing in the southwest part of Poland are at a lower risk: Silesian (7%), Lubusz, Opole, Lower Silesian (11%). The same value (11%) was observed in the Mazovian voivodeship as well. Voivodeship proved to have a different impact on the risk of living conditions poverty than on income poverty. The highest rates of living conditions poverty were recorded in Warmian-Masurian (12%), West Pomeranian (12%), Świętokrzyskie (11%) and Łódź (11%), and the lowest in Podlaskie (5%), Greater Poland (6%) and Silesian (7%). Poverty in terms of lack of budget balance more often affects the West Pomeranian households (18%). Other voivodeships with high shares of households experiencing difficulties with balancing their budgets included Lubusz (15%), Lodz (15%), Pomeranian (14%) and Warmian-Masurian (14%). The lowest values were reported in Subcarpathian (6%) and Podlaskie (7%).

### Correlation analysis

The relevance and magnitude of associations between cancer risk and socioeconomic variables varies greatly by cancer site (Table 4). Spearman’s correlation analysis revealed a statistically significant strong positive correlation between lung cancer risk and: (1) social isolation (rS = 0.65; *p* = 0.007), (2) two types of poverty: living conditions poverty (rS = 0.51; *p* = 0.04) and poverty following a lack of budget balance (rS = 0.66; *p* = 0.006), and (3) low/no involvement in religious activity (rS = 0.66; *p* = 0.005). A statistically significantly strong negative correlation was found between risk of lung cancer and: (1) high level of association-based social capital (rS = − 0.64; *p* = 0.008), and (2) high religiousness (rS = − 0.68; *p* = 0.004). After age and sex adjustment the Spearman’s correlation coefficients remained similar, with the exception of correlation with association-based social capital which was stronger after correcting for sex (rS = − 0.84; *p* = 0.0001). Adjustment for completeness of cancer registry strengthened the positive association between lung cancer incidence and low/no involvement in religious activity (rS = 0.85; *p* = 0.001), and made the negative associations between lung cancer incidence, family based social capital and friend- and neighbour-based social capital stronger and statistically significant (rS = − 0.561; *p* = 0.03 and rS = − 0.56; *p* = 0.03, respectively). After adjustment for completeness of cancer registry the correlations between lung cancer incidence, living conditions poverty and association-based social capital were no longer statistically relevant.

**Table 4:** Spearman’s correlation coefficients for the relationship between cancer incidence and socioeconomic variables: rS: crude Spearman correlation coefficients, partial rS: partial Spearman correlation coefficients adjusted for sex (1), age (2), and completeness of cancer registration (3)

Socioeconomic indicator	Cancer site					
	Lung		Breast		Colon	
	RS (*p*)	Partial rS (p)	RS	Partial rS (*p*)	RS	Partial rS (*p*)
High level of social isolation	0.65 (0.007)	(1) 0.66 (0.007)	0.36 (0.17)	(1) 0.39 (0.16)	0.195 (0.47)	(1) 0.186 (0.5)
		(2) 0.68 (0.006)		(2) 0.36 (0.18)		(2) 0.195 (0.5)
		(3) 0.68 (0.006)		(3) 0.42 (0.1(1)		(3) 0.31 (0.26)
Social capital						
Association-based	− 0.64 (0.008)	(1) − 0.84 (0.0001)	− 0.36 (0.17)	(1) − 0.39 (0.15)	− 0.3 (0.26)	(1) − 0.32 (0.25)
		(2) − 0.68 (0.006)		(2) − 0.36 (0.18)		(2) − 0.32 (0.25)
		(3) − 0.5 (0.057)		(3) − 0.31 (0.27)		(3) − 0.24 (0.4)
Family based	− 0.375 (0.15)	(1) − 0.36 (0.182)	− 0.3 (0.25)	(1) − 0.43 (0.11)	− 0.01 (0.96)	(1) − 0.17 (0.55)
		(2) − 0.286 (0.29)		(2) − 0.36 (0.22)		(2) − 0.19 (0.51)
		(3) − 0.561 (0.03)		(3) − 0.25 (0.37)		(3) − 0.17 (0.55)
Friend- and neighbour-based	− 0.38 (0.15)	(1) − 0.51 (0.051)	− 0.74 (0.001)	(1) − 0.64 (0.01)	− 0.56 (0.02 5)	(1) − 0.55 (0.03)
		(2) − 0.4 (0.14)		(2) − 0.74 (0.002)		(2) − 0.56 (0.03)
		(3) − 0.56 (0.03)		(3) − 0.76 (0.001)		(3) − 0.65 (0.01)
Low/no involvement in religious activity	0.66 (0.005)	(1) 0.68 (0.005)	0.53 (0.04)	(1) 0.53 (0.04)	0.17 (0.53)	(1) 0.14 (0.6)
		(2) 0.77 (0.001)		(2) 0.54 (0.04)		(2) 0.16 (0.56)
		(3) 0.85 (< 0.001)		(3) 0.53 (0.04)		(3) 0.43 (0.11)
High religiousness	− 0.68 (0.004)	(1) − 0.69 (0.005)	− 0.57 (0.02)	(1) − 0.59 (0.02)	− 0.28 (0.3)	(1) − 0.275 (0.3)
		(2) − 0.63 (0.012)		(2) − 0.64 (0.01)		(2) − 0.31 (0.26)
		(3) − 0.78 (0.001)		(3) − 0.53 (0.04)		(3) − 0.37 (0.17)
Poverty						
Income	− 0.04 (0.88)	(1) − 0.1 (0.67)	− 0.51 (0.04)	(1) − 0.28 (0.31)	− 0.01 (0.96)	(1) 0.042 (0.88)
		(2) − 0.047 (0.87)		(2) − 0.46 (0.09)		(2) 0.035 (0.9)
		(3) − 0.259 (0.35)		(3) − 0.49 (0.06)		(3) − 0.326 (.24)
Living conditions	0.51 (0.04)	(1) 0.48 (0.07)	− 0.01 (0.96)	(1) 0.001 (1)	0.063 (0.82)	1) 0.065 (0.81)
		(2) 0.49 (0.07)		(2) − 0.011 (0.97)		(2) 0.065 (0.82)
		(3) 0.44 (0.1)		(3) − 0.034 (0.9)		(3) − 0.072 (0.8)
In terms of dealing with the household budget	0.66 (0.006)	(1) 0.67 (0.006)	0.39 (0.13)	(1) 0.42 (0.12)	0.15 (0.58)	(1) 0.14 (0.62)
		(2) 0.66 (0.008)		(2) 0.39 (0.15)		(2) 0.16 (0.57)
		(3) 0.708 (0.003)		(3) 0.42 (0.12)		(3) 0.19 (0.5)

*p* < 0.05 was considered statistically significant

The risk of breast cancer was statistically significantly negatively related to: (1) income poverty (rS = − 0.51; *p* = 0.04), (2) high level of friend- and neighbour-based social capital (rS = − 0.74; *p* = 0.0009), and (3) high religiousness (rS = − 0.57; *p* = 0.02), and positively related to low/no involvement in religious activity (rS = 0.53; *p* = 0.04). After sex, age and completeness of data adjustment the correlations between breast cancer incidence and socioeconomic variables were not substantially modified, only correlation with income poverty became statistically insignificant.

The only statistically relevant for colon cancer was a negative correlation between colon cancer risk and high level of friend- and neighbour-based social capital (rS = − 0.56; *p* = 0.025). Adjustment for age, sex and data completeness produced fairly similar coefficients.

These results should be interpreted in light of relationships that may exist between the socioeconomic variables selected to the analysis. Theoretically these determinants have different meaning, but there are correlations between them, except for relationships between income poverty and other socioeconomic determinants, various forms of social capital, as well as social isolation and informal social capital (family-, friend- and neighbour-based social capital). Lack of correlation between income poverty and other poverty forms and strong correlation between living conditions poverty and poverty following a lack of budget balance (rS 0.7; *p* = 0.002) imply that a low income situation (determined by revenue) does not necessarily signify other poverty forms. Time factor is, therefore, extremely important, because poor living conditions and budget difficulties result from unfavourable income situation continuing for a longer period. The strong relationship with material situation (poverty following a lack of budget balance and living conditions poverty) observed for association-based capital and social isolation suggests a coexistence of these factors. All of these determinants were correlated with lung cancer risk. However, the question whether poverty caused social isolation and low level of association-based social capital, or if, conversely, social isolation and low level of association-based capital caused poverty, is still to be answered.

## Discussion

### Result summary and relation to other work

The findings of our study indicate that poor social relationships are linked to higher incidence of lung, breast and colon cancer in Poland. As it is currently uncertain whether a particular form of social relationships is more predictive than others, using several types of measures of social relationship and different types of cancer, our study allows for comparisons that have not been conducted before. Earlier studies from other countries reported a correlation between social isolation and cancer-specific mortality and less satisfactory prognosis among some cancer sites (Beasley et al. 2010; Pinquart et al. 2010; Ikeda et al. 2013; Hinzey et al. 2016; Lutgendorf et al. 2012; Kroenke et al. 2017). Furthermore, there is evidence that social integration is associated with lower rates of mortality (Holt-Lunstad et al. 2010). An analysis of results from 26 prospective studies revealed that the link between lack of social relations and cancer progression was most notable in breast cancer and not seen in remaining types of cancers (Nausheen et al. 2009). Relatively few studies have assessed the effects of social relations on cancer incidence directly. Early systematic review of longitudinal studies (Garssen et al. 2004) provided weak evidence that social relations influence cancer incidence and prognosis: seven studies proved that social relations were strongly associated with cancer initiation or progression, whereas seven others did not. In part, this may be because the type of social capital measures varied considerably among studies. A recently published Japanese study showed more significant risk of colorectal cancer incidence and mortality among men with low social support (Ikeda et al. 2013).

Regardless the important evidence linking low level of social capital relationships and social isolation to poor health outcomes, the causal mechanisms still remain not fully understood. It is suggested that social bonds impact health behaviour, partly because they influence, or even “control” our health habits and provide support which enables individuals to cope with stressors (Holt-Lunstad et al. 2010). Understanding the nature and extent of the correlation between social relations and cancer risk is of urgent importance, given evidence that social bonds in developed countries are declining.

We found that the risk of lung and breast cancer was positively correlated with low/no involvement in religious activity and negatively correlated with high religiousness. Literature review showed that at least 29 studies have examined relationship between religion/spirituality and different outcome of cancer (Koenig 2012). Over half of them (16) reported lower risk of developing cancer or a better prognosis in individuals who are more religious/spiritual, only 2 found inverse relationship (Koenig 2012). Of the 20 studies with the most rigorous methodology, 12 (60%) found an association between religion/spirituality and lower risk of cancer or better outcomes, and none reported a negative relationship. Results described above may be somewhat explained by the fact that involvement in religious activity boosts supportive social interactions and is associated with better health behaviours.

The site identified in our study as being linked with lower socioeconomic status is consistent with previous research which showed correlation between lung cancer incidence and higher poverty (Boscoe et al. 2014; Bryre et al. 2014; Singh et al. 2003). Results concerning the relations between breast cancer incidence and socioeconomic status are contradictory. Our findings are supported by the US studies, which indicate consistently higher incidence of breast cancer in lower poverty groups, with incidence rates rising more rapidly among low poverty than among high poverty populations (Singh et al. 2003; Conroy et al. 2017; Reynolds et al. 2005). The lack of social gradient in the incidence rate of breast cancer was observed in the French population (Bryere et al. 2014). The incidence rate of colorectal cancer was faintly or inconsistently linked to poverty rate (Singh et al. 2003; Boscoe et al. 2014; Bryere et al. 2014), which is in line with our findings.

As presented in our study, the correlation between socioeconomic status and cancer is complex and varies by cancer type. In general, cancer sites associated with behavioural risk factors, i.e., tobacco, alcohol, intravenous drug use, sexual transmission and poor diet tend to be linked to higher poverty (Boscoe et al. 2014; Singh et al. 2003). The trend in high incidence of breast and colon cancer among low poverty populations may be explained by a greater participation of the members of wealthier socioeconomic classes in screening programs. Additionally, well established breast cancer risk factors such as advanced age for first birth, hormone replacement therapy and specific diet behaviour which contributes to the development of colon cancer are more common in socioeconomically developed populations. This suggests that socioeconomic environment cannot be considered as a determining factor of cancer in a biological sense but since several cancer risk factors are associated with social inequalities, poverty is a “cause of the cause”.

### Limitations

Results of our study should be interpreted cautiously, bearing in mind limitations attributable to the ecological design of the study. The methodological problem fundamental in assuming individual associations based on group data (ecological fallacy) is well known and repeatedly discussed (Diez Roux 2004; Loney and Nagelkerke 2014). The major limitation of our study is the absence of individual socioeconomic characteristics and cancer risk. Variables were measured on voivodeship level making it impossible to adequately control the confounding factors, effect modifiers and mediators at an individual level In consequence, disparities documented in our study do not reflect the experience of an individual, they rather reveal a diversity of cancer incidence in geographic areas which are stratified in accordance to major social and economic determinants. To overcome this limitation a multilevel analysis operating both at an individual and contextual level is required. It is impossible to distinguish the individual and contextual effect of a variable using ecological design. For instance, by relating income poverty rate to lung cancer incidence it is hard to differentiate whether these differences are due to the effects of the income poverty level of an individual or to the contextual effects of the rate of income poverty. This indicator, although created through an aggregation of information on individual members of the group, might provide information distinct from their individual level analogue: rate of income poverty may be an indicator of neighbourhood-level factors possibly related to health and these factors may affect each member of the community, regardless of their individual level of income. However, from a public health perspective, the ecologic association may itself be of concern, regardless of whether it is confounded by individual-level variables or whether it results from contextual or compositional effects. Another limitation was the cross-sectional character of our study: we sought to quantify life course influences and cancer risk using indicators measured at a given point of time. However, it should be emphasized that indicators used in our study depend not only on the current, but also previous situation: for example, the current material situation, especially in terms of living conditions and the ability to maintain financial stability and to deal with the budget, depending on general material resources which may be related to both current and previous income. An unfavourable income situation, continuing for a longer period, fosters the accumulation of poor living conditions and budget difficulties. Indicators for social isolation, social capital and involvement in church/religious activity depend on long time behaviour, tradition and culture specific to given geographical regions and in this sense are good proxies to estimate exposure across one’s life course. Taking this into consideration, we can assume that measuring these indicators at a given point of time for individual voivodeships may provide information about lifetime exposure to these risk factors in specific geographical regions.

### Implications

Despite these limitations the main findings of our study support public health concerns over the implication of socioeconomic environment for cancer and suggest that addressing social capital and poverty may have a crucial role in preventing the leading causes of mortality in our country. Even though socioeconomic variables may not be the direct cause of cancer, they impact cancer risk indirectly through two main pathways: creating situations which give rise to risk factors (i.e., tobacco and alcohol use, unhealthy diet, lack of physical activity) and influencing health care accessibility, use of cancer screening programmes and effective treatments. National policy actions which can have an important effect on the magnitude of social inequalities in cancer incidence might significantly reduce the burden and disparities in various populations and geographic areas. Our study showed that the relevance and magnitude of associations between socioeconomic variables and cancer risk vary greatly by cancer site. It provides important additional information about how socioeconomic mechanisms work. Social and economic inequalities can be avoided; therefore their limitation constitutes an achievable goal and an ethical imperative.
